# Impact of the Extremities Positioning on the Set-Up Reproducibility for the Total Marrow Irradiation Treatment

**DOI:** 10.3390/curroncol30040309

**Published:** 2023-04-06

**Authors:** Nicola Lambri, Simone Leopoldo Antonetti, Damiano Dei, Luisa Bellu, Stefania Bramanti, Ricardo Coimbra Brioso, Carmelo Carlo-Stella, Isabella Castiglioni, Elena Clerici, Leonardo Crespi, Chiara De Philippis, Carmela Galdieri, Daniele Loiacono, Pierina Navarria, Giacomo Reggiori, Roberto Rusconi, Stefano Tomatis, Marta Scorsetti, Pietro Mancosu

**Affiliations:** 1 Radiotherapy and Radiosurgery Department, IRCCS Humanitas Research Hospital, Via Manzoni 56, 20089 Milan, Italy; 2 Department of Biomedical Sciences, Humanitas University, Via Rita Levi Montalcini 4, 20072 Milan, Italy; 3 Radiation Oncology Department, SS. Antonio e Biagio e Cesare Arrigo Hospital, 15121 Alessandria, Italy; 4 Department of Oncology and Hematology, IRCCS Humanitas Research Hospital, Via Manzoni 56, 20089 Milan, Italy; 5 Dipartimento di Elettronica, Informazione e Bioingegneria, Politecnico di Milano, 20133 Milan, Italy; 6 Department of Physics “G. Occhialini”, University of Milan-Bicocca, Piazza della Scienza 2, 20126 Milan, Italy; 7 Centre for Health Data Science, Human Technopole, 20157 Milan, Italy

**Keywords:** total marrow irradiation (TMI), total marrow lymph node irradiation (TMLI), patient positioning, reproducibility, volumetric modulated arc therapy (VMAT), radiotherapy (RT)

## Abstract

Total marrow (lymph node) irradiation (TMI/TMLI) delivery requires more time than standard radiotherapy treatments. The patient’s extremities, through the joints, can experience large movements. The reproducibility of TMI/TMLI patients’ extremities was evaluated to find the best positioning and reduce unwanted movements. Eighty TMI/TMLI patients were selected (2013–2022). During treatment, a cone-beam computed tomography (CBCT) was performed for each isocenter to reposition the patient. CBCT-CT pairs were evaluated considering: (i) online vector shift (OVS) that matched the two series; (ii) residual vector shift (RVS) to reposition the patient’s extremities; (iii) qualitative agreement (range 1–5). Patients were subdivided into (i) arms either leaning on the frame or above the body; (ii) with or without a personal cushion for foot positioning. The Mann-Whitney test was considered (*p* < 0.05 significant). Six-hundred-twenty-nine CBCTs were analyzed. The median OVS was 4.0 mm, with only 1.6% of cases ranked < 3, and 24% of RVS > 10 mm. Arms leaning on the frame had significantly smaller RVS than above the body (median: 8.0 mm/6.0 mm, *p* < 0.05). Using a personal cushion for the feet significantly improved the RVS than without cushions (median: 8.5 mm/1.8 mm, *p* < 0.01). The role and experience of the radiotherapy team are fundamental to optimizing the TMI/TMLI patient setup.

## 1. Introduction

Total body irradiation (TBI) is commonly adopted in conditioning regimes for allogeneic hematopoietic stem cell transplantation to help in eradicating tumor cells and provide a sufficient level of immunosuppression to prevent the rejection of donor hematopoietic cells. Generally, TBI treatments are performed with the patient standing or lying down at a large source-to-surface distance (SSD—approximately 4 m) to cover the entire patient’s body with a single large treatment field [1]. Therefore, dedicated equipment (e.g., couches, shields, dose attenuators to homogenize the dose) and larger bunkers than those usually used in radiotherapy (RT) are required. Randomized trials have demonstrated the efficacy of TBI compared to chemoconditioning only [2,3,4,5,6]. However, the late toxicity effects to critical organs drove the research towards alternative more targeted forms of irradiation, such as total marrow irradiation (TMI) or total marrow lymph-nodes irradiation (TMLI), to reduce the dose to healthy tissues while maintaining, or increasing, the dose to the hematological target [7].

TMI/TMLI was initially investigated using helical tomotherapy and intensity-modulated radiotherapy (IMRT) [8,9,10,11,12,13]. Afterward, several groups showed the technical feasibility of TMI/TMLI delivered with volumetric modulated arc therapy (VMAT) using C-arm linacs [14,15,16], and, more recently, a preclinical validation of VMAT-TMI/TMLI optimized using a ring gantry linac has been reported [17]. The advantage of all of these modulated techniques is that they are delivered using a standard isocentric RT approach with the patient lying down on a regular couch, which allows the use of common immobilization systems and image-guided RT for repositioning the patient.

To cover the TMI/TMLI clinical target volume (CTV), i.e., the whole bone marrow, the entire patient’s length has to be irradiated. Tomotherapy utilizes a continuous helical field focused along the treatment axis, with the treatment couch sliding through the gantry. On the other hand, IMRT and VMAT require multiple arcs and isocenters, as the maximum linac’s jaw aperture is 40 × 40 cm^2^. Furthermore, the TMI/TMLI delivery must be split into two parts, one for the upper part of the body in head-first supine (HFS), and one for the lower extremities in feet-first supine (FFS), because of the limited travel range (130–150 cm) of both the delivery system (i.e., helical tomotherapy, C-arm linac, and ring gantry linac) and computed tomography (CT) couches. Consequently, the door-to-door time of a TMI/TMLI treatment can exceed 1 h for all these delivery methodologies, thus increasing the chance of involuntary patient motion. Moreover, with C-arm and ring gantry linacs the use of multiple isocenters can produce unwanted over/under-dosage in the regions where fields belonging to two adjacent isocenters overlap. Therefore, in general, the patient immobilization system is essential throughout the whole RT process of TMI/TMLI to ensure accurate patient position reproducibility.

In recent studies, four different groups reported their clinical practice for patient immobilization. Bao et al. used a homemade dedicated immobilization system, which consisted of one all-body frame, one integrated vacuum-formed cradle, one upper limb fixator, and three personalized thermoplastic masks [18]. Haraldsson et al. [19] and Shahid et al. [17] used a whole-body vacuum cushion with a thermoplastic mask for, respectively, the head and shoulders, or the head alone. Finally, in our previous works, we described and evaluated a homemade 3-frame immobilization system with three personalized thermoplastic masks and two separate feet immobilizers [20,21].

A crucial aspect of the immobilization of a TMI/TMLI patient, common to all TMI/TMLI immobilization systems and delivery techniques, is the position of the extremities, which, through the joints, could experience large movements. Image-guided RT allows the performance of a rigid registration between the simulation CT and the online imaging acquired during the treatment session, privileging the matching of body structures at the expense of the extremities, which could thus receive an under/over-dosage. This issue may be particularly relevant for helical tomotherapy, as a single extended megavoltage CT (MVCT) is commonly acquired to determine a unique shift before the start of the treatment. While this approach can also be applied to IMRT and VMAT, more standard practice involves acquiring a kilo voltage cone beam CT (kV-CBCT) and performing a shift at each isocenter, thus reducing the risk of intra-fraction patient movements.

To the best of our knowledge, the impact of the patient’s extremities reproducibility for TMI/TMLI treatments has not yet been investigated in the literature. To this aim, in this study, we evaluated the reproducibility of TMI/TMLI patients’ extremities to find the best positioning and reduce unwanted movements which are not accounted for during a TMI/TMLI treatment.

## 2. Materials and Methods

### 2.1. Simulation and Target Volume Definition

Since 2010, more than 100 adult patients have been treated in our institute with TMI or TMLI employing VMAT, with a prescribed dose of 2 Gy (1 fraction) [22], following an internal protocol approved by the Institutional Ethics Committee of IRCCS Humanitas Research Hospital. According to the protocol, all patients were simulated on a Brilliance Big Bore CT system (Philips Health Care) in the supine position. Due to the CT scanning limit (130–150 cm), the total cranial-caudal (CC) length of a patient was acquired with two CT images reconstructed with a 5 mm slice thickness, one in HFS for the upper body and a second one in FFS for the lower extremities.

The upper-body CT scan extended from head to knees and was acquired in free breathing mode. The lower-extremities CT scan extended from the feet to the femoral heads. The acquisitions overlapped by ~20 cm (on the femurs) to ensure accurate registration between the two CT series for the creation of a robust field junction in the overlap region. The same immobilization device was used in both CT scans. Between the two acquisitions, the patient was taken off the couch, the immobilization frame was rotated to the feet-first position and the patient was placed back on the frame.

To provide an additional margin around the bone marrow, the TMI clinical target volume (CTV) was defined as the individual bones, with the exclusion of hands, mandible, and maxillary structures. The planning target volume (PTV) was defined as CTV plus an isotropic expansion of 2 mm. Furthermore, the whole chest wall was considered as part of the PTV to include the breathing motion of the ribs, and the bones of the extremities (i.e., arms and legs) were isotropically expanded by 10 mm to account for setup uncertainties and potential intra-fractional motion. For TMLI treatments, the spleen and lymph nodes plus an additional isotropic margin of 5 mm were included in the PTV.

### 2.2. Immobilization System

Patients were immobilized using a dedicated immobilization frame (ELSE Solutions s.r.l.), which has been presented in our previous studies [20,21]. The system is composed of 2 rectangular Plexiglas boards (80 × 60 cm^2^) and a specific head and neck board which accounts for the larger size of the shoulders. The frame thickness is 2 cm and is thus included in the calculation grid due to its non-negligible X-ray attenuation effect. Each board of the frame can be combined with the adjacent one via an interlocking shape. Two separate feet immobilizers are attached to the most caudal board. Each immobilizer can be regulated in both CC and lateral directions depending on the patient’s lower-extremities anatomy.

A graduated scale was printed on top of the boards to guarantee patient positioning reproducibility. Near the lateral edges of each board, several rows composed of 5 equally spaced pegs used to fix the thermoplastic masks were positioned at different heights in the CC direction. To best immobilize the patient, three masks were used for (i) the head and shoulders, (ii) the thoracic and pelvic region, and (iii) the lower extremities with a specific fixation within the legs. The upper extremities (hands and arms) were immobilized alongside the body inside the thermoplastic mask to ensure patient comfort and reproducibility.

### 2.3. Treatment Planning

All plans were delivered with VMAT and optimized for a Varian TrueBeam equipped with a Millennium multi-leaf collimator (Varian Medical Systems, Palo Alto, CA, USA). Plan optimizations were performed with the Eclipse (Varian Medical Systems, Palo Alto, CA, USA) treatment planning system (TPS). Isocenters’ positioning and field geometry was decided based on the planner’s experience [23]. For the upper-body plan, 4–6 isocenters for a total of ten full arcs (360°) were considered. Each arc overlapped with the adjacent ones for at least 2 cm on each side such that the differences in delivered dose distributions concerning planning due to small patient misalignment between isocenters were minimized [20]. For obese patients, the position of the arms was more than 20 cm from the medial axis and the maximum field aperture (40 cm) was not sufficient to provide adequate target coverage. Thus, two additional isocenters on the arms were required. In these situations, because the TPS allows optimizing at most 10 arcs at the same time during a single optimization, 4 isocenters were used to cover the torso, while 2 isocenters were used for the arms. An analogous approach for the lower-extremities plan was adopted (3 isocenters and 6 arcs).

### 2.4. Image-Guided Radiotherapy

Image-guided RT was performed with online CBCT for each isocenter before the delivery of the specific isocenter. The recommendation was to minimize shifts to 1–3 mm in the CC direction and to 3–5 mm in LR and AP directions, following the work of Mancosu et al. [20]. A radiation oncologist matched the CBCT to the simulation CT using a rigid registration based on bones, with an eventual minimal adjustment on soft tissues (<2 mm). The CBCT-CT matching prioritized the body structures, with no formal recommendation on the positioning of the patient’s extremities.

### 2.5. Study Design

Eighty TMI/TMLI patients treated between 2013 and 2022 were randomly selected from our clinical database. All patients in this study were treated with the same homemade immobilization frame, but the fixation method used for the extremities varied case by case, depending on the patient’s anatomy, availability of equipment, and the RT technologist’s (RTTs) expertise. Furthermore, the delivery process was subject to additional variability due to the non-standard treatment, the small number of patients per year (~10), and the frequent turnover of RTTs (not dedicated to this specific pathology).

**Figure 1:**
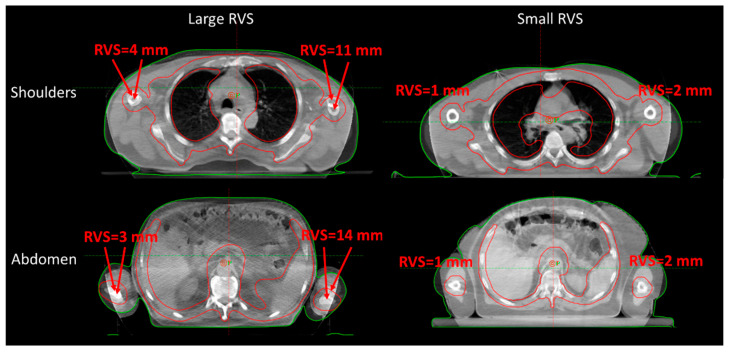
Representative CBCT-CT matching was performed for the shoulders (**top**) and abdomen (**bottom**) isocenters. The RVS was measured as the maximum center distance between the bone marrow of the extremities. Large (**left**) and small (**right**) RVS values are reported. RVS = residual vector shift.

**Figure 2:**
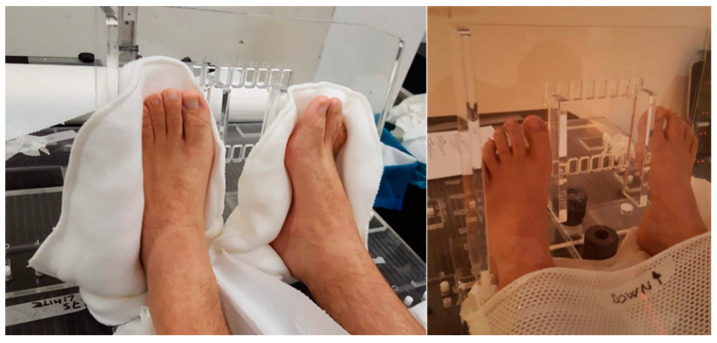
Feet positioning with (**left**) or without (**right**) a personal cushion.

Therefore, all CBCTs were evaluated considering: (i) the CBCT-CT online vector shift (OVS), computed from the CC, AP, and LR shifts, that best matched the two series; (ii) the residual (bi-dimensional) vector shift (RVS) which would still be needed after the online matching to reposition the patient’s extremities in the original simulated position, see Figure 1; (iii) the CBCT-CT agreement using a qualitative rating with range 1–5 (from poor to optimal). Patients were subdivided according to the extremities immobilization methods: (i) arms either leaning on the immobilization frame to maximize patient comfort, or above the body to minimize the lateral field of view and facilitate the plan optimization; and (ii) lower extremities with or without a personal cushion for feet positioning. Such cushions, shown in Figure 2, were available when discarded from other cranial treatments.

### 2.6. Statistical Analysis

Statistical analysis was performed using Python v3.10.4 with NumPy v1.22.4 and SciPy v1.8.1 libraries. The Mann-Whitney test for independent samples was considered, with a value of *p* < 0.05 as statistically significant.

## 3. Results

A total of 629 CBCT were analyzed. Table 1 reports the median values and interquartile range (IQR) of the OVS between CBCT-CT and the RVS for the extremities, divided per anatomical region covered by each isocenter (see Table S1 in the Supplementary Materials for the shifts in CC, AP, and LR direction). The greater median OVS for isocenters on the abdomen/arms (4.7 mm—IQR of (3.0, 6.4 mm)) and feet (4.4 mm—IQR of (2.6, 5.4 mm)), as well as the extremities’ median RVS (>7 mm), confirm that the extremities were the anatomical region subject to larger positioning variability, thus making the CBCT-CT matching more problematic. The overall median OVS was 4.0 mm, with an IQR of (2.4, 6.0) mm. A larger overall median RVS for the left and right extremities was observed, with a value of 4.8 mm and 5.1 mm, respectively. Twenty-four percent of the RVS of the extremities was >10 mm (CTV-PTV margin = 10 mm), with a major impact on the dose distribution in 0.6% of cases.

The mean qualitative rating improved over the years, from 3.6 in 2013 up to 4.5 in the 2019–2022 period (see Figure S1 in the Supplementary Materials). Table 2 summarizes for each anatomical site the OVS grouped by qualitative rating. Good overall quality was observed, with only 1.6% of cases ranked below level 3 (i.e., 10 CBCTs with a rating of 2 and none with a rating of 1). No significant differences were found between rating groups, except for rating 3 vs. 4 of the abdomen/arms (median OVS: 5.4 mm vs. 4.4 mm, respectively), and rating 4 vs. 5 of the hip (median OVS: 4.4 mm vs. 3.2 mm, respectively).

**Figure 3:**
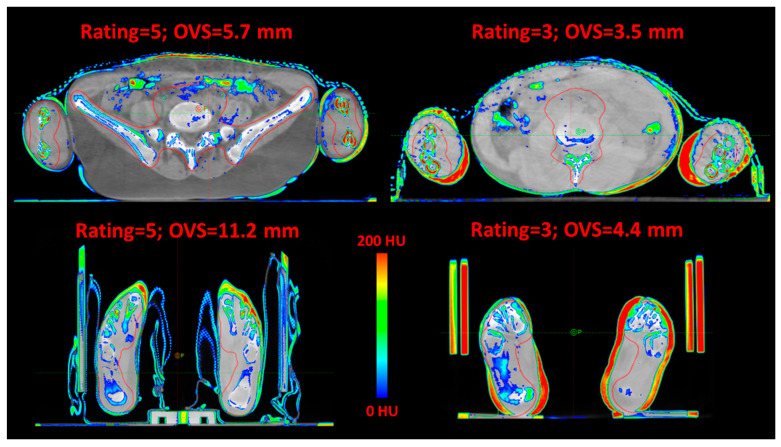
Four representative CBCT-CT matchings of upper and lower extremities. Color wash was used to highlight differences >200 HU. The qualitative rating and OVS are reported. OVS = online vector shift.

Figure 3 shows four representative CBCT-CT matchings of the upper and lower extremities, demonstrating that large online shifts did not necessarily correspond to lower qualitative ratings.

The specific analysis concerning the patient immobilization method for the extremities is reported in Table 3 (see Table S2 in the Supplementary Materials for the shifts in CC, AP, and LR direction). No significant differences were found in OVS between arms leaning on the frame vs. above the body (median: 4.5 mm vs. 5.2 mm), and in the use of a personal cushion to fix the feet (median: 4.1 mm vs. 4.5 mm). A significant improvement was observed for the RVS of the arms, with a median decrease from 8.0 mm to 6.0 mm for both the left and right extremities (*p* < 0.05 and *p* < 0.02, respectively). A similar fraction of RVS > 10 mm was observed for arms leaning on the frame vs. above the body, with 17.6% vs. 15.6% and 15.6% vs. 18.5% for, respectively, the left and right extremities. The use of a personal cushion to fix the feet greatly improved the RVS than without a cushion, with a median RVS of 1.8 mm vs. 8.5 mm (*p* < 0.01), and 8.4 mm vs. 2.6 mm (*p* < 0.01), for left and right extremity, respectively. Accordingly, the fraction of RVS > 10 mm was much smaller with the feet cushion than without cushion, with 5% vs. 23.8% and 2.5% vs. 22.5% for the left and right extremities, respectively.

## 4. Discussion

In this study, we investigated both quantitatively and qualitatively the CBCT-CT matching for each isocenter of 80 retrospective TMI/TMLI patients treated with VMAT at our Institution between 2013 and 2022. This work was the result of an RTT master’s thesis in advanced techniques of oncological RT conducted at Humanitas University (Italy).

The main focus of this paper was to investigate and ensure the reproducibility of the patient’s extremities positioning in TMI/TMLI treatments, as this is a common issue that affects all types of TMI/TMLI deliveries. Due to the large treatment volume, it is fundamental to evaluate also the “peripheral” parts of the body that may experience large movements. Despite being small, these regions constitute part of the target and therefore require complete coverage.

The quantitative assessment was carried out by measuring the CBCT-CT OVS needed to best reposition the patient at each isocenter, revealing that abdomen/arms and feet isocenters were subject to larger variability (i.e., largest online shifts). The observed overall median OVS was 4.0 mm (see Table S1 in the Supplementary Materials for the shifts in CC, AP, and LR direction). For comparison, Bao et al. reported average setup corrections of 1.3 ± 0.7 mm, 2 ± 1 mm, 1.1 ± 0.8 mm, in, respectively, the CC, AP, and LR direction [18]. Patients were treated with helical tomotherapy, and 4 mega-voltage CTs were acquired in different regions to check the patients’ whole body alignment during treatment. The authors used a homemade dedicated immobilization system, which consisted of one all-body frame, one integrated vacuum-formed cradle, one upper limb fixator, and three personalized thermoplastic masks. In another recent study, Shadid et al. performed a preclinical validation of TMI/TMLI delivered with Halcyon linac (Varian Medical Systems, Palo Alto, CA, USA), reporting average shifts of −1 ± 2 mm, 3 ± 3 mm, and −4 ± 5 mm, in, respectively, the CC, AP, and LR direction [19]. Patients were immobilized using a whole-body vacuum cushion and a thermoplastic mask for the head. A CBCT was acquired for each of the 3 plans needed to deliver the treatment.

The qualitative evaluation was achieved by rating the CBCT-CT matching on a scale of 1–5 (from poor to optimal). The only significant reduction in OVS that corresponded to an increase in rating was found for abdomen/arms and hip isocenters, possibly due to the presence of elbows and hands which can experience large movements, thus making the patient repositioning more challenging.

All patients in this study were treated with the same homemade immobilization frame, but the fixation method used for the extremities varied case by case, depending on the patient’s anatomy, availability of equipment, and the RTT’s expertise. Thus, to evaluate the best-suited immobilization system, we computed the RVS of the patient’s extremities concerning the immobilization method used to fix them. The analysis revealed that arms leaning on the frame (alongside the patient’s body) significantly reduced the median RVS for the left and right extremities, compared to arms above the body. A possible explanation is that the first method is more comfortable for the patient, which can more easily maintain the same position as the simulation CT. However, one drawback is the larger field of view, which, in the case of a large patient, could potentially make necessary the use of specific isocenters for the arms due to the maximum Linac’s jaw aperture of 40 cm. Another significant decrease in the median RVS was found when using a personal cushion to immobilize the patient’s feet instead of the raw frame (see Figure 2). Specifically, a significant reduction was observed in the median RVS for, respectively, the left and right extremities. Again, this result can be explained because the patient’s comfort was improved. Nonetheless, the cushion could not be used for all patients because it was available only when discarded from other cranial treatments.

To the best of our knowledge, this is the first study that investigated the patient’s extremities reproducibility in TMI/TMLI treatments. We think that these technical data regarding patient positioning are of current significance, particularly as new preclinical advancements in TMI/TMLI require implementation into clinical studies [24,25]. Our results demonstrated that careful consideration should be posed when deciding the immobilization method to use for the extremities. Specifically, a more comfortable patient positioning was associated with better reproducibility (lower RVS), although the lateral field of view needed to irradiate the target (abdomen/arms) could become large. The role of the whole RT team, including radiation oncologists, medical physicists, and RTTs, is thus fundamental for choosing the immobilization method which can best reposition the patient during a TMI/TMLI treatment. Furthermore, despite the frame design can appear optimal for immobilization, patient comfort should be considered as well on the same level of importance for its creation. Therefore, with their expertise and direct contact with the patient, RTTs should guide the development and upgrading of devices for patient immobilization.

Classical TBI is delivered at large SSD in a non-standard condition, therefore requires specific in-vivo measurements using thermo-luminescent dosimeters (TLD) or optical surface luminescence dosimeters (OSLD) to verify the actual delivered dose. An advantage of the TMI/TMLI treatment is that it is performed with a regular couch in isocentric conditions. Given these standard treatment conditions, no specific systematic studies are needed: for all patients, we follow a quality assurance (QA) process recommended by international guidelines (AAPM-TG218—[26]) to evaluate the deliverability of the RT plan before the start of the treatment. Furthermore, specifically for TMI/TMLI, in our center, we have performed a non-systematic study using GafChromic EBT3 films for bi-dimensional in-vivo dosimetry verification of partially overlapping arcs with different isocenters [27]. The dose maps measured with the EBT3 films were compared with the corresponding calculations along the patient immobilization frame. The gamma agreement index (GAI) with a 5% dose difference and 5 mm distance to the agreement was computed, and values > 95% were observed for all patients.

At our institute, since October 2010, TMI/TMLI has been delivered using the VMAT technique. We emphasize that the patient positioning issues of TMI/TMLI are the same also for other intensity-modulated techniques, such as tomotherapy and IMRT. The immobilization systems used with helical tomotherapy are very similar to the one used in this study. Thus, our analysis can be beneficial to centers optimizing TMI/TMLI with techniques other than VMAT.

In this study, intra-fractional organ motion was not investigated. Due to the large target volume, it is challenging to perform a systematic evaluation of intra-fraction motion as a different part of the target may exhibit different motion movements. To minimize the impact of intra-fraction motion, a CBCT scan was acquired for each isocenter (i.e., every 5–10 min).

This work is part of the AuToMI project, which was created to guide centers which are going to introduce TMI/TMLI in their clinic, as well as to implement automated tools to streamline the TMI/TMLI treatment planning [28,29,30]. The results presented here will be used to design an improved version of the immobilization frame, which will include a dedicated cushion for the feet. Consequently, the expected smaller patient’s shifting and residual positioning uncertainty could allow a reduction of the 10 mm CTV-PTV expansion on the extremities. These margins were obtained from our first treated patients to ensure that the CTV (i.e., bones of the extremities) was covered with the prescription dose for nearly all cases.

## 5. Conclusions

The analysis of retrospective CBCT-CT matchings allowed us to evaluate which immobilization method is best suited for TMI/TMLI patient setup and to optimize the procedure. All future patients will be positioned with arms leaning on the frame, alongside the body, and feet immobilized with a personal cushion. The role and experience of the whole RT team in TMI/TMLI patient setup are fundamental for choosing the immobilization method which can best reposition the patient.

## Figures and Tables

**Table 1:** Median values of the OVS between CBCT-CT and the RVS for the extremities, for each isocenter position. IQR values are reported in parentheses.

Isocenter Position	OVS [mm]	Left Extremity RVS [mm]	Right Extremity RVS [mm]
**Head**	3.8 (2.3, 5.1)	-	-
**Shoulders**	2.8 (1.6, 4.2)	2.9 (0, 6.2)	5.1 (0, 8.0)
**Abdomen/Arms**	4.7 (3.0, 6.4)	7.0 (4.5, 10.0)	7.3 (3.3, 9.8)
**Hip**	4.1 (2.5, 6.4)	3.8 (0, 8.9)	5.1 (1.2, 8.9)
**Legs**	4.1 (2.4, 6.3)	2.0 (0, 5.8)	2.1 (0, 6.0)
**Feet**	4.4 (2.6, 6.4)	7.5 (3.0, 11.0)	7.5 (3.6, 10.4)
**Total**	4.0 (2.4, 6.0)	4.8 (0, 8.0)	5.1 (0, 8.4)

Legend: OVS = online vector shift; RVS = residual vector shift.

**Table 2:** Median values of the OVS between CBCT-CT grouped by qualitative rating. IQR values are reported in parentheses. Values for “rating 2” are reported explicitly due to the small sample size. No cases of “rating 1” were found.

	OVS (mm)					
Rating	Head	Shoulders	Abdomen/Arms	Hip	Legs	Feet
**2**	-	-	3.5, 7.3, 17.6	4.7, 6.1, 22.5	2.4, 13.8	1.7, 3.0
**3**	2.7 (1.9, 3.6)	2.7 (1.8, 3.2)	5.4 * (4.6, 7.5)	4.8 (3.0, 8.7)	4.5 (2.3, 6.1)	4.3 (2.6, 7.1)
**4**	5.8 (3.9, 10.8)	3.7 (2.0, 5.4)	4.4 * (2.7, 5.8)	4.4 † (2.9, 6.7)	3.6 (2.2, 5.3)	4.7 (2.9, 7.4)
**5**	3.8 (2.4, 5.1)	2.7 (1.3, 3.7)	4.0 (3.3, 5.8)	3.2 † (2.0, 5.0)	4.3 (2.7, 7.1)	3.8 (2.6, 4.8)

Legend: OVS = online vector shift. Symbols (*, †) denote statistically significant differences.

**Table 3:** Median values of the OVS between CBCT-CT and the RVS for the extremities, grouped by immobilization method. IQR values are reported in parentheses.

Extremities Immobilization Method	OVS (mm)	Left Extremity RVS (mm)	Right Extremity RVS (mm)
**Arms leaning on the frame**	4.5 (3.1, 6.2)	6.0 † (3.0, 9.0)	5.9 * (0, 8.9)
**Arms above the body**	5.2 (3.0, 6.5)	8.0 † (5.0, 10.0)	7.5 * (5.0, 11.0)
**With feet cushions**	4.1 (2.8, 5.3)	1.8 ‡ (0, 6.3)	2.6 ** (0, 7.3)
**Without feet cushions**	4.5 (2.4, 7.2)	8.3 ‡ (5.9, 11.0)	8.2 ** (5.9, 12.0)

Legend: OVS = online vector shift; RVS = residual vector shift. Symbols (*, **, †, ‡) denote statistically significant differences.

## Data Availability

The data presented in this study are available on request from the corresponding author.

## Supplementary Materials

The following supporting information can be downloaded at: https://www.mdpi.com/article/10.3390/curroncol30040309/s1, Figure S1: Mean qualitative rating over the years; Table S1: CC, AP, LR online shifts; Table S2: CC, AP, LR immobilization methods online shifts.
