# Supplementary material for: Impact of the Extremities Positioning on the Set-Up Reproducibility for the Total Marrow Irradiation Treatment
Source: Curr Oncol. 2023 Apr 6;30(4):4067–77. doi: 10.3390/curroncol30040309 (PMC10136565; doi:10.3390/curroncol30040309)
Supplement: Supplementary file 1 [file curroncol-30-00309-s001.zip › curroncol-2286766-supplementary.pdf]

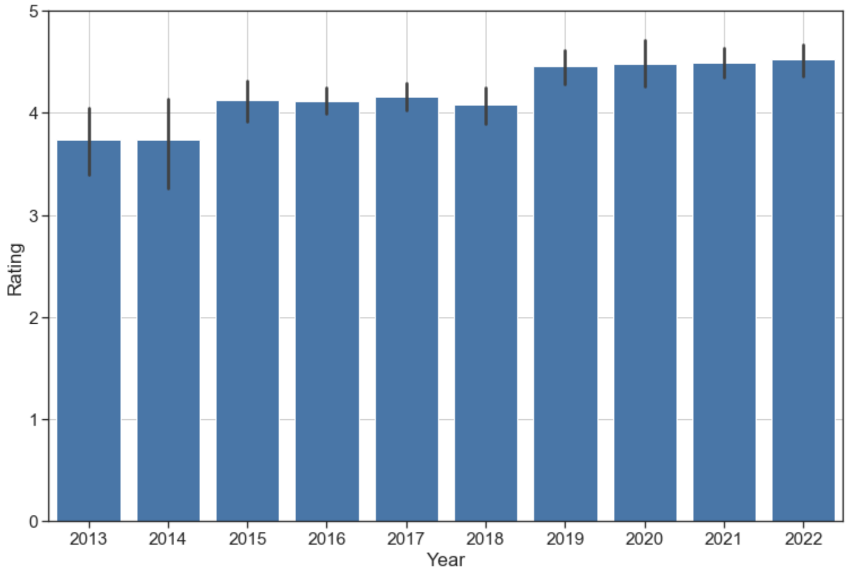

Figure S1. Mean qualitative rating over the years. Bars indicate the standard deviation.

Table S1. CC, AP, LR isocenters online shifts. Median values of the online shift between CBCT-CT in CC, AP, and LR direction for each isocenter position. IQR values are reported in parentheses.

	CC online shift [mm]	AP online shift [mm]	LR online shift [mm]
**Head**	-0.2 (-2.2, 1.2)	-0.6 (-1.9, 1.2)	-0.3 (-1.9, 1.1)
**Shoulders**	0 (0, 0.1)	-0.3 (-2.0, 0.7)	0 (-1.1, 1.4)
**Abdomen/Arms**	0 (0, 1.0)	-1.1 (-3.0, 0)	1.1 (-2.0, 4.2)
**Hip**	0 (-0.1, 0.7)	-1.5 (-3.7, 0.1)	0 (-2.1, 1.6)
**Legs**	0 (-0.1, 0.2)	0.1 (-1.3, 1.8)	0 (-2.3, 2.1)
**Feet**	0 (0, 1.8)	1.5 (0, 2.8)	-0.4 (-2.1, 1.1)
**Total**	0 (-0.1, 0.7)	0 (-2.1, 1.5)	0 (-2.1, 2.2)

Table S2. CC, AP, LR immobilization methods online shifts. Median values of the online shift between CBCT-CT in CC, AP, and LR direction, grouped by immobilization method. IQR values are reported in parentheses.

Extremities immobilization method	CC online shift [mm]	AP online shift [mm]	LR online shift [mm]
**Arms leaning on the frame**	0 (0, 1.3)	-0.9 (-2.6, 0)	0 (-2.8, 3.2)
**Arms above the body**	0 (0, 0.6)	-1.1 (-2.8, 0)	1.6 (-0.6, 4.3)
**With feet cushions**	0.1 (-0.6, 1.8)	1.5 (0.2, 2.6)	-0.4 (-2.0, 2.5)
**Without feet cushions**	0 (0, 1.7)	1.5 (0, 2.9)	-0.5 (-2.0, 0.8)
